# Genetic Association Mapping via Evolution-Based Clustering of Haplotypes

**DOI:** 10.1371/journal.pgen.0030111

**Published:** 2007-07-06

**Authors:** Ioanna Tachmazidou, Claudio J Verzilli, Maria De Iorio

**Affiliations:** 1 Department of Epidemiology and Public Health, Imperial College London, United Kingdom; 2 Department of Epidemiology and Population Health, London School of Hygiene and Tropical Medicine, United Kingdom; University of Chicago, United States of America

## Abstract

Multilocus analysis of single nucleotide polymorphism haplotypes is a promising approach to dissecting the genetic basis of complex diseases. We propose a coalescent-based model for association mapping that potentially increases the power to detect disease-susceptibility variants in genetic association studies. The approach uses Bayesian partition modelling to cluster haplotypes with similar disease risks by exploiting evolutionary information. We focus on candidate gene regions with densely spaced markers and model chromosomal segments in high linkage disequilibrium therein assuming a perfect phylogeny. To make this assumption more realistic, we split the chromosomal region of interest into sub-regions or windows of high linkage disequilibrium. The haplotype space is then partitioned into disjoint clusters, within which the phenotype–haplotype association is assumed to be the same. For example, in case-control studies, we expect chromosomal segments bearing the causal variant on a common ancestral background to be more frequent among cases than controls, giving rise to two separate haplotype clusters. The novelty of our approach arises from the fact that the distance used for clustering haplotypes has an evolutionary interpretation, as haplotypes are clustered according to the time to their most recent common ancestor. Our approach is fully Bayesian and we develop a Markov Chain Monte Carlo algorithm to sample efficiently over the space of possible partitions. We compare the proposed approach to both single-marker analyses and recently proposed multi-marker methods and show that the Bayesian partition modelling performs similarly in localizing the causal allele while yielding lower false-positive rates. Also, the method is computationally quicker than other multi-marker approaches. We present an application to real genotype data from the CYP2D6 gene region, which has a confirmed role in drug metabolism, where we succeed in mapping the location of the susceptibility variant within a small error.

## Author Summary

Genetic association studies offer great promise in dissecting the genetic contribution to complex diseases. The underlying idea of such studies is to search for genetic variants along the genome that appear to be associated with a trait of interest, e.g., disease status for a binary trait. One then proceeds by genotyping unrelated individuals at several marker sites, searching for positions where single markers or combinations of multiple markers on the paternally and maternally inherited chromosomes (or haplotypes) appear to discriminate among affected and unaffected individuals, flagging genomic regions that may harbour disease susceptibility variants. The statistical analysis of such studies, however, poses several challenges, such as multiplicity and false-positives issue, due to the large number of markers considered. Focusing on case-control studies, we present a novel evolution-based Bayesian partition model that clusters haplotypes with similar disease risks. The novelty of this approach lies in the use of perfect phylogenies, which offers a sensible and computationally efficient approximation of the ancestry of a sample of chromosomes. We show that the incorporation of phylogenetic information leads to low false-positive rates, while our model fitting offers computational advantages over similar recently proposed coalescent-based haplotype clustering methods.

## Introduction

Genetic association studies have emerged as a powerful tool for dissecting the genetic contribution to complex, common diseases. Their main goal is to identify inter-individual genetic variants, mostly single nucleotide polymorphisms (SNPs), which show the strongest association with the phenotype of interest, either because they are causal or, more likely, statistically correlated or in linkage disequilibrium (LD) with an unobserved causal variant(s). Univariate analyses that test each marker for association with the phenotype can be inefficient, as they do not take into account the patterns of LD among markers as opposed to multi-marker or haplotype-based approaches.

Haplotype-based analyses are promising and their use is supported by results from recent studies that suggest that the human genome consists of block-like regions of ancestrally conserved chromosomal segments, whose boundaries are defined by recombination hotspots [1–3]. The main difficulty with a haplotype-based approach is that, for a large number of SNPs, there may be many haplotypes, usually a few common and several rare ones. One solution is to model all rare haplotypes as a single “exposure” group, but this approach could lead to loss of information.

An alternative approach to sensibly reducing the number of haplotypes considered is to cluster structurally “similar” haplotypes, as they are more likely to carry the same susceptibility allele and therefore have similar associated risk [4]. The rationale behind this approach is that haplotypes that inherit a causal mutation, e.g., case haplotypes for a dichotomous trait, tend to also inherit alleles at markers nearby due to LD. Therefore, case haplotypes are expected to be more similar around the causal locus compared to control haplotypes. Hence, similar haplotypes are grouped together in homogeneous clusters, within which disease risk is assumed constant [4]. A key issue with such haplotype clustering methods is the choice of the metric used to determine how similar one haplotype is to another. The similarity metric can be, for example, the proportion of SNPs at which two haplotypes are the same [5], or it can exploit the ancestral relationships of haplotypes by adopting the notion that if the causal mutation has occurred only once then haplotypes share a common ancestry at that point [6–11].

One recently proposed clustering method is that of Waldron et al. [11]. They modify the ideas of Molitor et al. [8] by looking for only one cluster with the highest disease risk haplotypes, and by modifying the similarity score to account for population allele frequencies and to allow allele mismatches. In particular, Waldron et al. [11] define a hypothetical ancestral haplotype (namely the cluster centre) from which the members of the cluster are thought to have descended, and they measure the similarity of the centre with each observed haplotype around a putative causal locus. The similarity metric is calculated for all windows containing the putative location. Each window score is the sum of the SNP scores and the final score for each unique haplotype is taken to be the maximum window score. The window with the maximum score is the part of the ancestral haplotype that the haplotype inherited. The cluster is defined to consist of all haplotypes whose similarity score exceeds some threshold. The ancestral haplotype, the causal locus, the penalty parameter for allele mismatches, and the threshold are random variables and are updated with a Markov Chain Monte Carlo (MCMC) algorithm.

Clustering approaches can be thought of as an empirical approximation to the more formal coalescent approach, which is promising for LD mapping [12], as the coalescent is more likely to infer a better approximation to the evolutionary history of mutations of a set of haplotypes. In fact, the genealogy of a sample of haplotypes contains the patterns of genetic diversity of the distinct haplotypes, with putative disease mutations embedded within. Several approaches based on the coalescent have been developed for fine-scale mapping [13,14]. However, most of these methods are effective only for a small number of markers and individuals.

The coalescent assumes that the variation in haplotypes can be described only by their mutational history. However, to approximate the shared ancestry among haplotypes more accurately, a fine-mapping approach may need to account for recombination. This can be achieved using methods that consider ancestral recombination graphs (ARG) [15, 16], but their computational complexity is still high.

In this paper, we propose a Bayesian partition model [17] to cluster haplotypes according to their associated level of risk by exploiting evolutionary information. The method is computationally fast and can handle large datasets with many markers and/or subjects. Bayesian partition models have been used in genetic association studies by Seaman et al. [18] for highly polymorphic candidate genes and by Molitor et al. [8] and Morris [19,20] for candidate genes or small candidate regions. We focus on candidate gene regions with densely spaced markers and assume that a perfect phylogeny holds over short chromosomal lengths in the region. The perfect phylogeny assumption implies that each SNP has arisen as a result of a single ancestral mutation. Recombination, parallel mutations, or back mutations can cause the perfect phylogeny assumption to be violated. The distance used for the clustering method has an evolutionary interpretation, as sequences are clustered together depending on the time to their most recent common ancestor in the genealogy. In particular, we proceed by splitting the chromosomal region of interest into sub-regions or windows where the perfect phylogeny assumption holds. Focusing on case-control studies, at each step of the MCMC algorithm we select a window, i.e., a perfect phylogeny, and we then partition the haplotype space into disjoint clusters on the basis of the relative ages of the markers in the selected window. Each cluster is then assigned a specific risk. Potentially, haplotypes can be clustered on the basis of any tree and each SNP has, a priori, a positive probability to be a cluster centre. The number and centres of the clusters are both assumed unknown, a priori. Our approach is fully Bayesian and we obtain posterior samples of quantities of interest, sampling over the space of possible partitions. We are particularly interested in the posterior probability of each SNP being a cluster, since high values correspond to markers or locations where case and control haplotypes are best separated, suggesting the presence of a disease susceptibility variant in the region. We assess the performance of the proposed method in a simulation study by comparing it with single locus analysis; to the haplotype-based method of Waldron et al. [11], as implemented in the software HAPCLUSTER; and to the ARG-based method of Minichiello and Durbin [16], implemented in the software Margarita. We consider various simulation scenarios differing in genetic relative risk, minor allele frequency of the causal allele, number of cases and controls, disease model, marker density, and recombination rate. Results indicate that the proposed method performs similarly in localizing the causal allele while yielding lower false-positive rates. Also, the method is computationally faster than other multi-marker approaches. We also apply the proposed method to real genotype data from the *CYP2D6* gene region, which has been shown to be associated with drug metabolism [21], and we succeed in mapping the location of the susceptibility variant within a small error.

## Results

### Simulation Studies

We investigated the performance of the proposed method using simulated case-control data under different scenarios. Results were compared to those obtained from the univariate Fisher's exact test of association at each SNP maker and those using the HAPCLUSTER algorithm [11]. The ARG-based Margarita [16] was only run on the default scenario as defined below because of computational time constraints. We choose HAPCLUSTER as a representative of alternative haplotype-clustering methods since Waldron et al. [11] found (in simulation studies) that it performs better than other similar methods such as BLADE [7] and DHSMAP [6]. They also found that their distance metric outperformed those of Durrant et al. [10] and Yu et al. [9]. An alternative ARG-based method is that of Zöllner and Pritchard [15]. However, in trial runs we found that it is not computationally feasible for such an extensive simulation study.

We used the software FREGENE [22] to simulate two pools of 20,000 haplotypes, corresponding to a uniform or variable recombination rate, spanning a 1-Mb chromosomal region. The population with constant recombination rate was simulated from the simple Wright-Fisher model with recombination and mutation rate equal to 2.3 × 10^−8^ and 1.1 × 10^−8^ per site per generation, respectively. The second population was simulated with recombination hotspots. We assumed that 60% of all recombination events take place in recombination hotspots, which occur on average every 200 kb and are 2 kb in length. Also, 1% of the genome was assumed to consist of hotspots. The recombination rate within hotspots was 6.56 × 10^−7^ per site per generation, and 4.44 × 10^−9^ between hotspots [22]. The mutation rate was 2.3 × 10^−8^ per site per generation.

To reflect ascertainment bias, we draw markers from the set of SNPs having minor allele frequency (MAF) larger than 1%. From these markers, 1,000 (or 340 depending in the SNP density chosen) SNPs were selected with probability proportional to *p*(1 − *p*), where *p* is the allele frequency of a marker in the sample, to reflect an extra ascertainment bias towards markers with two common alleles and to give 1- (or 3-) kb average SNP density. A causative locus was then selected at random with allele frequency between *p* − 0.005 and *p* + 0.005, where *p* was in a range between 0.02 and 0.3. Then, for each pair of randomly sampled haplotypes, the case/control status was assigned according to either an additive or dominant disease model for the genotypes at the causal site assuming a disease prevalence *K* equal to 1% while the genetic relative risk of the heterozygote genetic relative risk (GRR[Aa]) varied between 1.2 and 2.4. Specifically, if *f_i_* is the penetrance function given *i* copies of the causal allele, *i* = 0, 1, 2, and GRR(Aa) = *r* = *f*_1_/*f*_0_, then following the liability model used in Tzeng [23] and assuming HWE, we have *f*_0_ = *K*/(1 − 2*p* + 2*pr*) and *f*_2_ = 2*rf*_0_ − *f*_0_ for an additive disease model, and *f*_0_ = *K*/(1 − 2*p* + 2*pr* + *p* ^2^ *− rp* ^2^) and *f*_2_ = *f*_1_ for a dominant one. Pairs of haplotypes were sampled with replacement from the 20,000 haplotypes until *N* cases and *N* controls were obtained. Thus, each case (control) individual contributed two case (control) haplotypes to the analysis. The sample size of cases and controls *N* also varied between 200 and 2,000.

Next, we removed the causal allele from the dataset and, using the algorithm described in Materials and Methods, we found the perfect phylogenies in the dataset. The average number of gene trees was 200 and the average number of SNPs in a gene tree was four. Using the SEQ2TR and the TREEPIC software of Griffiths [24], we obtained the relative ages of the mutations in the different phylogenies. We assumed a Beta(1,1) prior for the haplotype risks implying that, a priori, each observed haplotype has a 0.5 risk of disease.

The MCMC algorithm was run for 100,000 iterations with a burn-in of 10,000 iterations for 50 datasets across different combinations of the simulation parameters. We define the “default” scenario as that corresponding to having *N* = 1,000 cases and controls simulated with variable recombination rate under an additive disease model with 1.6 GRR(Aa), a SNP density of 1 kb, and a causal allele with 5% MAF.

The computing time for a dataset of 1,000 markers and 4,000 haplotypes was approximately 23 min (14 min to construct the phylogenies and 9 min to run the algorithm) on an Intel Xeon 3.40GHz processor with 2 Gb of memory. The corresponding computing time for HAPCLUSTER was 24 min. Note that while HAPCLUSTER is written in C++, the proposed method is implemented in R. As mentioned earlier, we compare the results from Margarita only under the default simulation scenario. To run Margarita on a single dataset of 1,000 markers and 4,000 haplotypes, we split the data into overlapping windows of 200 markers and then run the algorithm separately on each window, as suggested by Minichiello (personal communication). This resulted in five windows for a single dataset. Each window took 15–16 h to run with 10,000 permutations on 100 ARGs on a high computing cluster of 2.66GHz Xeon 5150 CPUs, making an exhaustive comparison of the two approaches impractical. An R package [25] called BETA (Bayesian Evolutionary Tree based Association analysis) implementing the method described in this article is available upon request from IT (ioanna.tachmazidou03@ic.ac.uk).

### One Liability Allele

**Figure 1:**
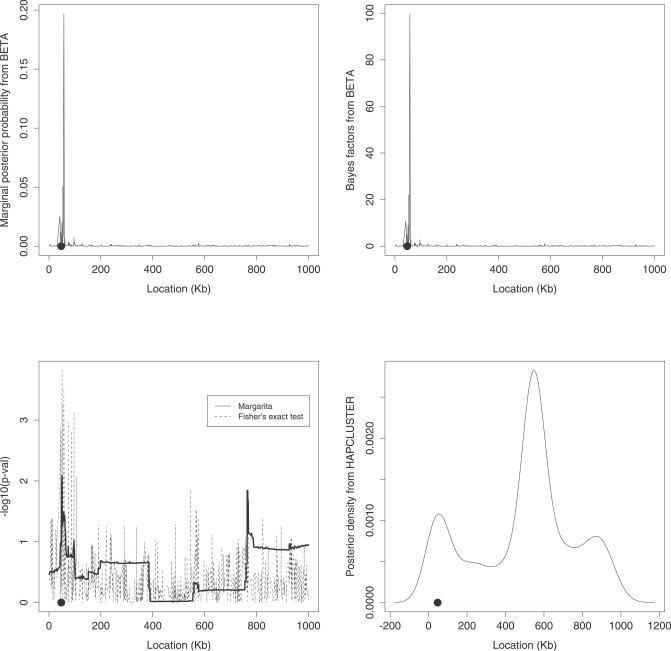
Results from BETA, Margarita, HAPCLUSTER and Fisher's Exact Test from a Single Dataset with One Susceptibility Allele under the Default Scenario Marginal posterior probability of association from BETA (top left), Bayes factor in favour of association at each marker from BETA (top right), *p*-values from Margarita and Fisher's exact test (bottom left), and posterior density of location from HAPCLUSTER (bottom right), where the dot on the x-axis indicates the position of the susceptibility mutation.

The results from a single simulated dataset under the default scenario are shown in Figure 1, where the dot on the x-axis indicates the position of the single susceptibility mutation. For the proposed method, the marginal posterior probability of association, i.e., the probability of each SNP being a cluster centre, and the Bayes factor in favour of association at each marker are shown. We also report the (log)*p*-values from Margarita and Fisher's exact test, and the posterior density of location from HAPCLUSTER. The estimate of the causal mutation is based on the marker with the minimum *p*-value (when using the single locus test and Margarita), the maximum Bayes factor (BETA), or the mode of the posterior distribution of location (HAPCLUSTER). For this dataset, all methods identified a marker within 10 kb of the true causal allele except for HAPCLUSTER (502-kb distance). The association signal is however notably clearer under the proposed method.

**Figure 2:**
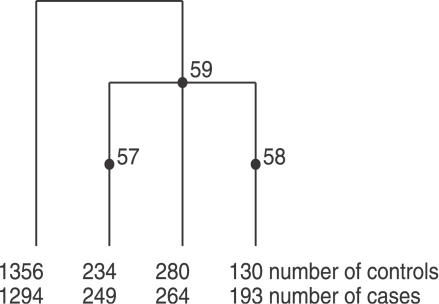
Perfect Phylogeny with the Highest Posterior Probability of Containing the Susceptibility Allele At the bottom of each branch we report the case and control multiplicities of each unique haplotype in the tree.

The same dataset contained 208 perfect phylogenies and Table 1 reports the posterior probability and Bayes factor of a tree carrying the causal locus, in which the numbers in brackets is the tree (all remaining trees had posterior probability less than 0.015). The true causal allele was embedded within tree 7 with marker S_43_ the closest to it. The posterior mode of the distribution for the number of clusters was two, including the “null” cluster (explained in the “Bayesian partition model” section of Materials and Methods), and SNP S_58_, which belonged to tree 10, had the highest marginal posterior probability of being a cluster centre. All marginal probabilities larger than 0.01 and corresponding Bayes factors are given in Table 2. The physically closest SNP to the true susceptibility allele in the table is S_47_, also embedded within tree 7. Figure 2 shows the perfect phylogeny with the highest posterior probability of containing the susceptibility allele (tree 10), together with the case and control multiplicities of each unique haplotype in the tree.

**Table 1:**
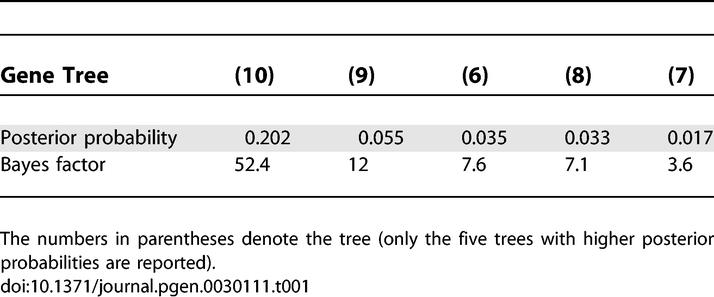
Posterior Probability of Each Gene Tree Carrying the Liability Allele and the Corresponding Bayes Factor in Favour of Association with the Disease

**Table 2:**
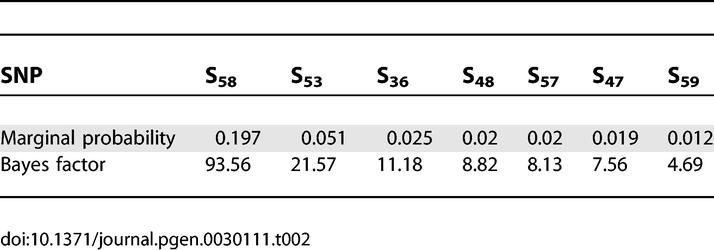
Marginal Posterior Probability of Each SNP Being a Cluster Centre and the Corresponding Bayes Factor (Only Those SNPs with Posterior Probabilities Greater than 0.01 Are Reported)

### Two Causal Alleles

**Figure 3:**
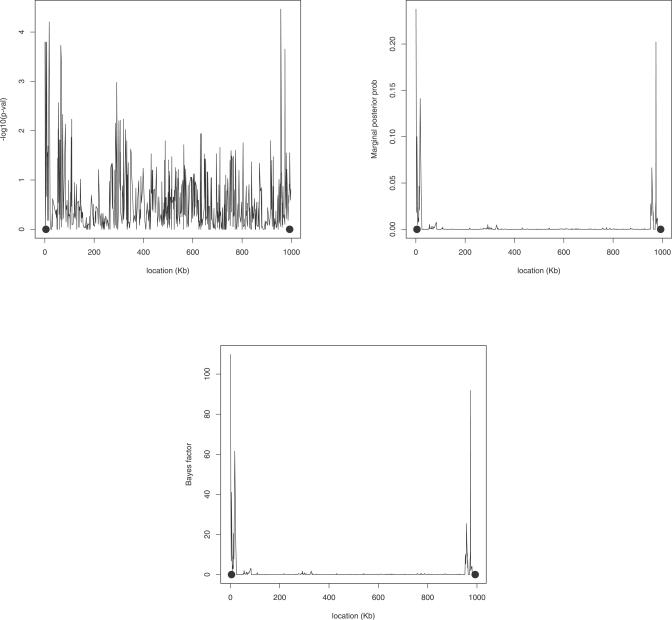
Results of Fisher's Exact Test and BETA from a Single Dataset with Two Susceptibility Alleles *p*-Values from Fisher's exact test for single-marker disease association (top left), the marginal posterior probability of association from BETA (top right), and the Bayes factor in favour of association at each marker from BETA (bottom centre), where the dots on the x-axis indicate the positions of two susceptibility mutations.

The proposed approach is not limited to the case of a single variant in a single candidate region. Figure 3 shows the results from a simulated dataset in which two liability alleles in separate regions (possibly corresponding to two separate candidate regions) contribute independently to disease susceptibility. The results reported correspond to genotype data for 200 cases and controls simulated assuming a variable recombination rate, an additive disease model, SNP density of 1 kb, MAF of causal alleles of 10–15%, and GRR(Aa) = 3. In total there were 184 perfect phylogenies with the two liability alleles belonging to trees 2 and 183. In Figure 3, the dots on the x-axis indicate the positions of the two susceptibility mutations. For this particular dataset, the Bayesian model appears to perform better than the single locus analysis, both in terms of location error and in reducing noisy associations. Trees 1 and 179 had the highest posterior probability of carrying the causal alleles, and the posterior mode of the distribution for the number of clusters was three, including the “null” cluster (see “Bayesian partition model” section of Materials and Methods). The distances between the loci with the highest marginal posterior probabilities of being cluster centres and the true locations of the susceptibility alleles were 4 kb and 19 kb, while the corresponding distances for the SNPs with the two smallest *p*-values were 13 kb and 36 kb. The advantage of the proposed method is likely due to the fact that we fully exploit the LD information around the causal alleles, incorporating the evolutionary information through the perfect phylogeny assumption.

### Performance Comparison

Tables 3–6 report the distances from the true location of the liability variant, together with its standard error for our method, HAPCLUSTER, and the single locus Fisher's exact tests under the different simulation scenarios. In each case, results shown are averages over 50 simulated datasets. The location of the causal allele is estimated by the SNP with the minimum *p*-value for Fisher's exact test and Margarita, by the posterior mode using a kernel density estimate for HAPCLUSTER, and by the SNP with the maximum Bayes factor or marginal posterior probability of being a cluster centre for the proposed model. For BETA, we report results both when the number of clusters is random and when it is fixed at two. Although the former assumption is more flexible, fixing the number of clusters to two is more sensible if, a priori, one expects only one causal mutation in the candidate region. Also, in the latter case, results are more directly comparable with those from HAPCLUSTER, which assumes only two clusters. In each table, the results under the default scenario are shown for ease of comparison. The average distance from the true location for Margarita over 50 replicates under the default simulation scenario is reported in Table 7, in which PERM *p*-value is the markerwise *p*-value calculated by permutation, EVD *p*-value is the markerwise *p*-value calculated by fitting an extreme value distribution, and EXP *p*-value is the experimentwise *p*-value calculated by permutation, as given by Margarita. Overall, there are no significant differences among the methods in terms of localization error. Figures S1–S14 show typical outputs from each of the methods considered under the default scenario. Note that for BETA, results are from the general version with a random number of clusters, as for all graphs shown. In the Supporting Information, we also report results of performance comparison over 100 datasets simulated under alternative scenarios (separated in Tables S1 and S2 depending on the MAF of the causal SNP).

**Table 3:**
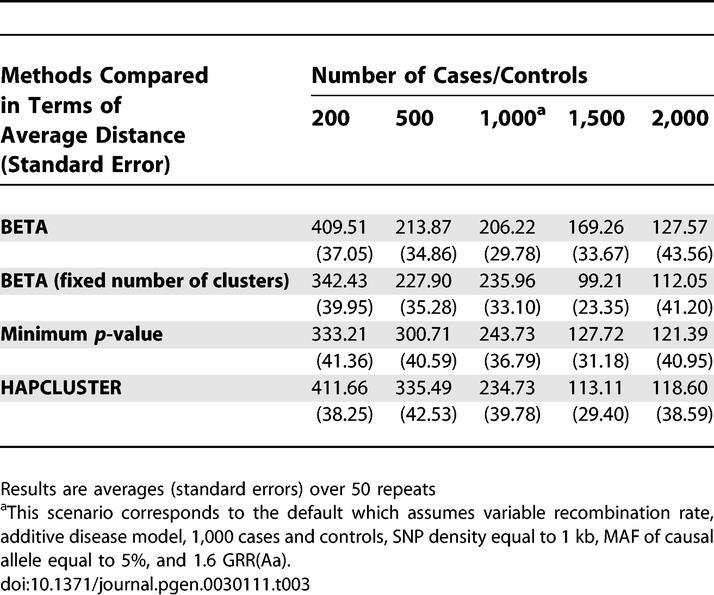
Average Location Errors (in kb) for BETA, HAPCLUSTER, and Single Locus Analysis for Different Number of Cases and Controls with All Other Simulation Parameters Set at Default Values

**Table 4:**
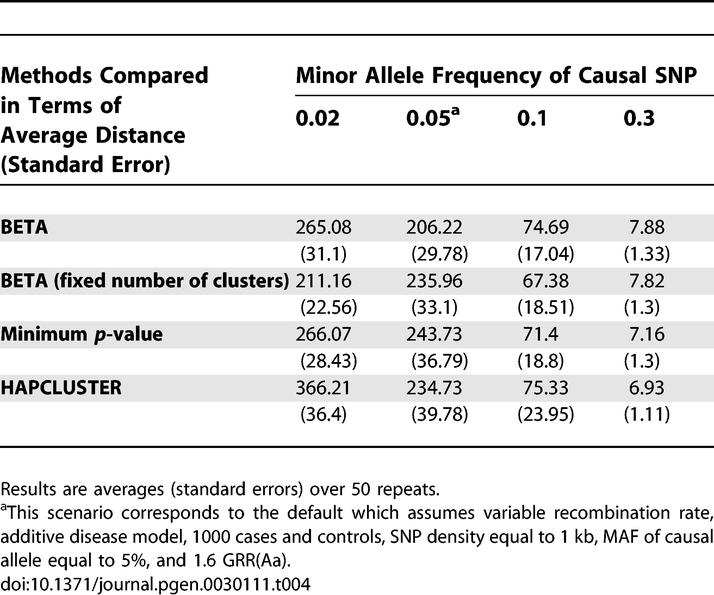
Average Location Errors (in kb) for BETA, HAPCLUSTER, and Single Locus Analysis for Different Allele Frequencies of the Causative Variant with All Other Simulation Parameters Set at Default Values

**Table 5:**
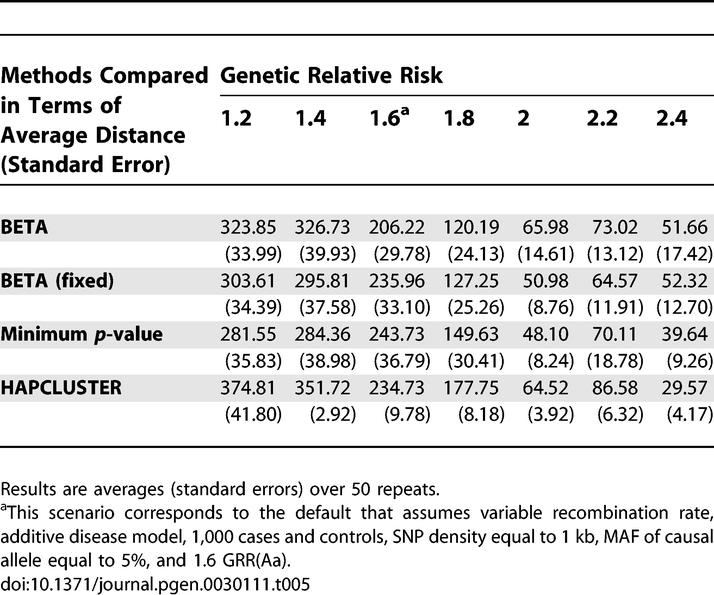
Average location errors (in kb) for BETA, HAPCLUSTER and single locus analysis for different genotype relative risks with all other simulation parameters set at default values

**Table 6:**
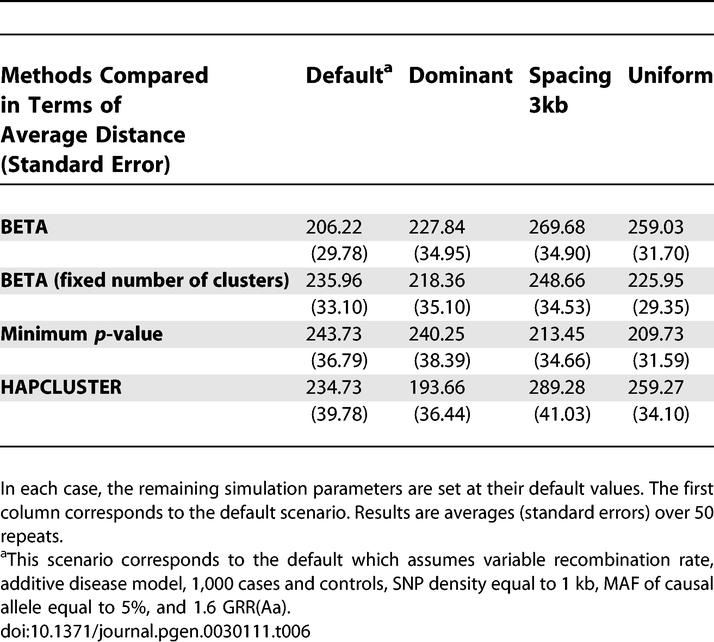
Average Location Errors (in kb) for BETA, HAPCLUSTER, and Single Locus Analysis for a Dominant Disease Model, SNP Density of 3 kb, or Uniform Recombination

**Table 7:**
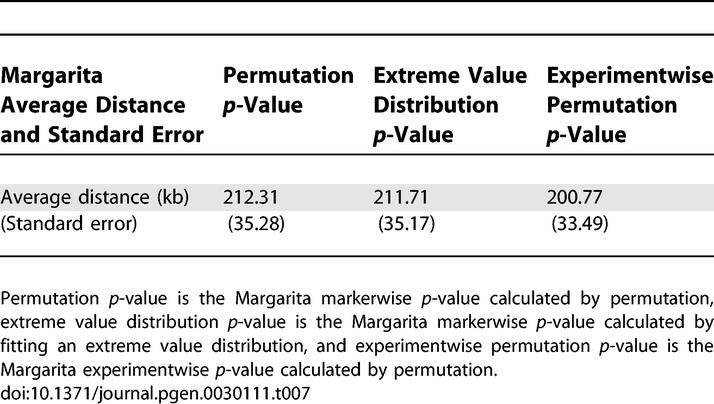
Average Distance (in kb) from the True Location of the Liability Loci Together with Its Standard Error for Margarita over 50 Replicates under the Default Simulation Scenario

**Figure 4:**
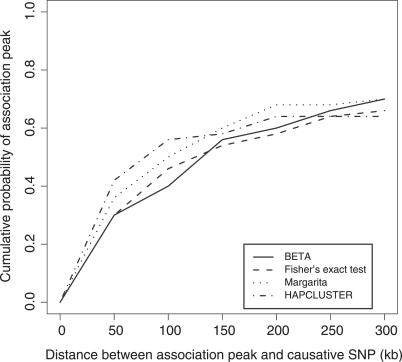
Cumulative Distribution of Distances between the Association Peak and the Causal SNP Analysis of 50 datasets simulated under the default scenario, namely variable recombination rate, additive disease model, 1,000 cases and controls, SNP density of 1 kb, MAF of causal allele 5%, and 1.6 GRR(Aa).

Similarly, there were no major differences in the distribution of the distances of the estimated and true location of the susceptibility allele for the different methods. Figure 4 plots the cumulative probability that the identified location is within some distance from the true location, over the 50 replicates and the default scenario. For reasonable location errors, the methods perform equally, with HAPCLUSTER possibly showing a slight advantage.

**Figure 5:**
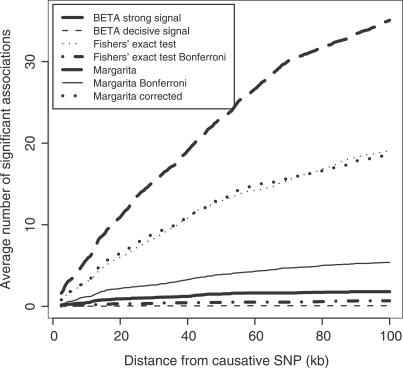
Average Number of Significant Associations within an Interval around the Causal SNP Analysis of 50 datasets simulated under the default scenario, namely variable recombination rate, additive disease model, 1,000 cases and controls, SNP density 1 kb, MAF of causal allele 5%, and 1.6 GRR(Aa). For “BETA strong signal” and “BETA decisive signal,” we consider markers with Bayes factors ≥10 and ≥150, respectively. For “Fisher's exact test” and “Fisher's exact test Bonferroni” we consider markers with *p*-values ≤0.05 and the Bonferroni-adjusted value respectively. For Margarita we consider the markerwise *p*-values calculated by permutation, while “Margarita Bonferroni” and “Margarita corrected” correspond to *p*-values corrected for multiple testing using Bonferroni and permutations, respectively.

On the other hand, the advantage of the proposed approach is evident when considering the number of false-positive associations over replicates, as well as the clarity in association signals. To quantify the latter, we consider a window around the causal SNP and calculate the average number of significant associations within that window across the 50 replicates. Results are shown in Figure 5. For BETA and single-marker tests, we report results from using two different significance thresholds, namely a Bayes factor in favour of association larger than or equal to 10 or 150 (corresponding to a strong or decisive signal, [26]) or a *p*-value smaller than or equal to 0.05 or the Bonferroni-adjusted value (0.05 divided by the number of markers in each dataset), respectively. For Margarita, we consider the markerwise *p*-values calculated by permutation, while “Margarita Bonferroni” and “Margarita corrected” correspond to *p*-values corrected for multiple testing using Bonferroni and permutation, respectively. Results for HAPCLUSTER are not reported, as this software does not provide markerwise estimates of measures of association.

The average number of associations found by BETA with a threshold of 10 for the Bayes factor remains stable as the distance increases and is lower than that given by all other methods apart from the single-marker Fisher's exact test results using a conservative Bonferroni adjustment. The latter, however, still yields a noisier signal than BETA under the more stringent threshold of 150 for the Bayes factor (bottom two lines in Figure 5).

**Figure 6:**
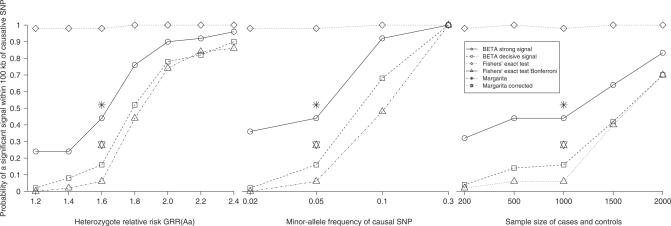
Power for a Range of Models Probability of a significant signal within 100 kb of the causal allele. Each point on the x-axis corresponds to 50 datasets under each of the simulation parameters while keeping the rest at their default values. The two points that do not belong to a line correspond to the default scenario for Margarita markerwise *p*-values calculated by permutation and Margarita experimentwise *p*-values calculated by permutation. For “BETA strong signal” and “BETA decisive signal” we consider markers with Bayes factors ≥10 and ≥150, respectively.

To compare the power of the different methods, we define a window of 100 kb on either side of the causative allele and calculate the proportion of the 50 replicates yielding a significant association within the window, as in Minichiello and Durbin [16]. The significance of a signal is assessed using the rules described in the previous paragraph. Figure 6 shows the probability of detecting a significant association within 100 kb of the causal SNP under various scenarios and over the 50 replicates. In each plot, we vary a simulation parameter along the x-axis while assuming default values for the remaining ones. As mentioned earlier, Margarita was run only for the default scenario. We were unable to obtain results from HAPCLUSTER, as this method does not give markerwise measures of association. From the results in Figure 6, BETA using the strong rule has more power than both the single locus approach and Margarita (default scenario only) with multiplicity-corrected results by permutation, and slightly less power than plain Margarita. Uncorrected single locus test is the most powerful approach, having, however, the worst performance in terms of false positives.

**Figure 7:**
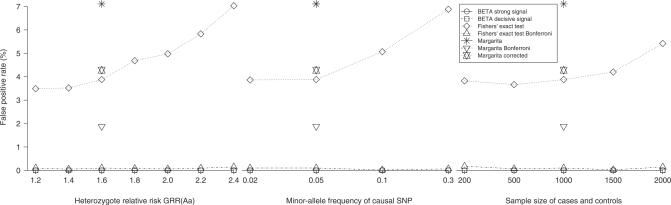
Mean False-Positive Rates (%) for Various Models Each point on the x-axis corresponds to 50 datasets under each of the simulation parameters while keeping the rest at their default values. The three points that do not belong to a line correspond to the default scenario for Margarita markerwise *p*-values calculated by permutation with or without Bonferroni correction, and Margarita experimentwise *p*-values calculated by permutation.

As noted earlier, an advantage of the proposed approach is the ability to remove much of the noisy associations. To investigate this further, we calculated the false positive rate from BETA and compared the results with the analogous quantity for Margarita and the univariate analysis. Specifically, given threshold *p*-values for Margarita and Fisher's exact test and threshold Bayes factors for BETA, we defined as false positives *M_f_p__*, those markers with smaller p-values or larger Bayes factors lying outside a window of 100 kb either side of the causal site [27]. For each dataset with *M* markers, the false positive rate is then *M_f_p__*/*M*. Figure 7 shows the mean false positive rates over the replicates and for different scenarios. The threshold values for the Bayes factors and the *p*-values are the same as in the previous analyses. For Margarita, the three points correspond to the default scenario and markerwise *p*-values calculated by permutation or experimentwise *p*-values calculated by permutation. The false-positive rates for BETA are very low under all simulation scenarios. Under the default scenario, BETA controls the false positives much better than Margarita. Results for HAPCLUSTER are not reported, since, as mentioned earlier, the method does not provide markerwise measures of association. Note that the choice of a 100-kb window is arbitrary; a 200-kb window was also used (unpublished data), which did not alter the conclusions about false positives.

Finally, we constructed 50 datasets under a null model of no disease association, and we calculated the false-positive rate. For the univariate analysis, this was 4.048% (*p*-value ≤0.05) and 0 when using Bonferroni correction, while BETA resulted in a false-positive rate of 0.138% (Bayes factor ≥10) and 0.002% (Bayes factor ≥150). Therefore, the proposed model appears to be reliable in confirming association.

### Application to CYP2D6 Data

The CYP2D6 gene on Chromosome 22q13 has a known role in drug metabolism, with multiple polymorphisms of CYP2D6 gene causing a recessive poor drug metaboliser phenotype. Hosking et al. [21] genotyped 1,018 individuals at 32 SNP markers across a 890-kb region flanking the CYP2D6 gene. From the 1,018 individuals, 41 were predicted to have the poor metaboliser phenotype and were thus treated as cases. This dataset has been used by Morris et al. [28], Maniatis et al. [29], Waldron et al. [11], and Verzilli et al. [27] to test their proposed LD mapping methods. Hosking et al. [21] reported a 390-kb region of significance around CYP2D6, Morris et al. [28] gave a 95% posterior confidence interval of 185 kb, and Maniatis et al. [29] yielded a 172-kb support interval, while Waldron et al. [11] and Verzilli et al. [27] refined it to 160 kb and 79 kb, respectively.

We used PHASE [30] to resolve ambiguous haplotype pairs for each individual. The pair for each individual was chosen at random according to the posterior probability of the haplotype pair provided by PHASE, and the resulting dataset was analysed as phase-known haplotype data. To investigate the effect of phase uncertainty, we repeated the above procedure ten times to obtain ten independent datasets, and ran the proposed method separately on each of these datasets.

Since the average SNP density for this dataset is 30 kb, we used a geometric prior distribution on the number of SNPs of each tree with parameter *p* equal to 0.98 (see “Model specification” section of Materials and Methods). An interpretation of this approach is that there is prior belief that the causal allele lies in a single-marker tree. Moreover, we fixed the number of clusters to be two, i.e., we expect a single causal location.

**Figure 8:**
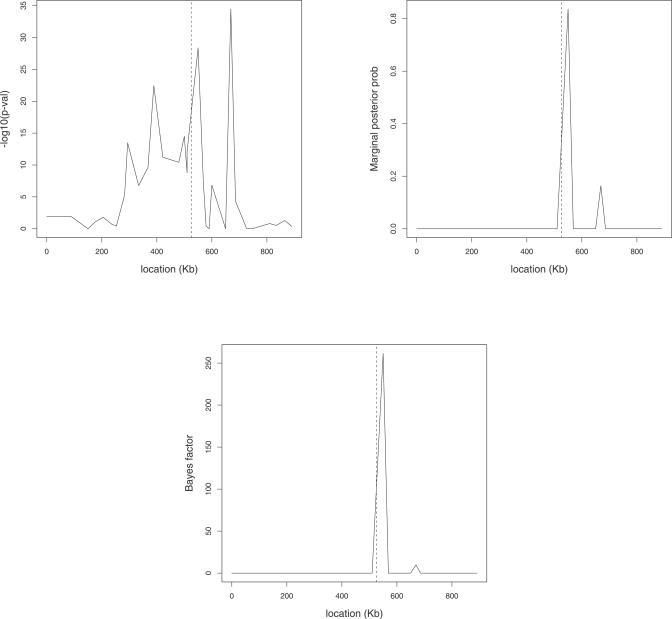
Results of Fisher's Exact Test and BETA Using the CYP2D6 Dataset *p*-Values from Fisher's exact test for single marker-disease association (top left), the marginal posterior probability of association (top right) and the Bayes factor in favour of association at each marker (bottom centre) from the CYP2D6 gene region, where the vertical line on the x-axis indicates the location of CYP2D6.

Each dataset consisted of 26 perfect phylogenies except for one that had 27. Most of the datasets resulted in 23 trees that contained a single SNP, two trees with two SNPs, and one tree with five SNPs. In all analyses, only gene trees 17, 18, 19, and 20 resulted in a non-zero posterior probability of carrying the liability allele (with an average of 0.75, 0.08, 0.15, and 0.02, respectively). Table 8 reports the marginal posterior probabilities and Bayes factors of each SNP being a cluster centre (averaged over the ten analyses), and Figure 8 shows *p*-values from Fisher's exact test for single-marker disease association, the marginal posterior probability of association and the Bayes factor in favour of association at each marker (again averaged over the ten analyses), where the vertical line on the x-axis indicates the location of CYP2D6. The results suggest strong evidence that marker 19 at 550 kb is the closest marker to gene CYP2D6 (at 525.3 kb), which leads to a location error of 24.7 kb. All ten analyses resulted in the same 95% credible interval of 119 kb. The same credible interval was given from all ten datasets analyzed by the general BETA version (where the number of clusters is random). In this case, in all ten imputed datasets, the posterior mode of the distribution for the number of clusters was two, including the “null” cluster (explained in the “Bayesian partition model” section of the Materials and Methods). The credible interval obtained by BETA compares favourably with the supporting intervals reported by other authors mentioned above.

**Table 8:**
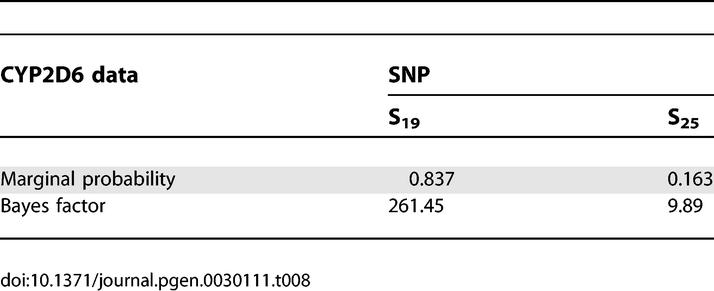
Marginal Posterior Probability of Each SNP Being a Cluster Centre and the Corresponding Bayes Factor for the CYP2D6 Data (All Remaining Markers Have Zero Posterior Probability)

## Discussion

We have presented a Bayesian method to perform an evolution-based association analysis using haplotype data. Haplotype data capture the genetic variation among individuals in a population, and their use in genetic association studies can potentially increase the power to locate susceptibility variants [31]. Our approach is based on the construction of rooted gene trees over small genetic regions. Although gene trees do not represent the exact history of haplotypes, they offer a sensible and computationally efficient approximation of the ancestry of a sample of chromosomes. The proposed algorithm is particularly suited for densely genotyped regions and can be applied to the analysis of single candidate genes, multiple candidate genes, or larger candidate regions. The performance of the proposed method has been compared with single-locus analyses and with recently proposed multi-locus methods in simulation studies. Results indicate that BETA performs similarly in localizing a causal allele, but leads to lower false-positive results. Moreover, it offers computational advantages over alternative multi-marker methods. In an application to real data from the CYP2D6 region, we are able to map the location of a susceptibility variant within a small error.

The proposed model is flexible and computationally efficient. It makes no assumptions about the disease model and allows modelling of multiple putative variants. Moreover, it can be easily extended to handle a continuous phenotype, and work is ongoing to apply it to genetic association studies with a survival outcome.

We have also presented a simplified version of the proposed method, in which we restrict the number of clusters to two, which is equivalent to looking for a single marker that best separates cases from controls. This is appropriate when we suspect a single susceptibility allele in the region of interest. In this way, we remove some variability, since we fix one of the parameters, leading to improved performance. Although, in general, the version of BETA with a random number of clusters is more flexible and realistic, we recommend using both versions and comparing the results.

The incorporation of environmental covariates in the model could be made possible by assuming, for instance, a cluster-specific probit regression. However, this is likely to be computationally demanding. Moreover, in our presentation of the method, we have assumed that the haplotypes are inferred from the genotypes with certainty. Although haplotype reconstruction is more reliable with dense markers and regions of strong LD, phase uncertainty ideally should be incorporated into the analysis. For instance, a fairer comparison with univariate analysis should probably involve simulating genotypes and then running our method on estimated haplotypes. Haplotype reconstruction programs, such as PHASE [30], output the posterior probabilities of haplotype pairs for each genotype, and we could randomly select the haplotype pair of an individual according to these posterior probabilities. This is the approach we used in the application to the real data from the CYP2D6 region. Alternatively, we could add a further Metropolis-Hastings (M-H) step and sample from the different haplotype reconstructions and perform the rest of the analysis (as described in Materials and Methods) given the chosen phase.

## Materials and Methods

Our Bayesian partition modelling involves two core analytical steps. We first split the genomic region under study into windows of high LD; this is done by sequentially constructing perfect phylogenies over the region of interest with window boundaries, which are deterministically defined by the locations where the perfect phylogeny assumption breaks down. Once the set of windows and corresponding trees have been identified, the Bayesian partition model searches through trees to identify those, if any, in which the corresponding set of haplotypes appear to form clusters that discriminate cases from controls, thus possibly harboring a causal variant. These two steps are described in detail next.

### Perfect phylogeny and gene trees.

Over small genomic regions, where LD is strong and recombination is low, it is reasonable to assume that haplotypes have evolved according to a perfect phylogeny [24]. Assuming nonrecurrent point mutations (in which case the infinitely-many-sites model holds), we can construct a unique tree that describes the mutation history of a sample of haplotypes. The tree is a representation of the haplotype data and it is useful to think of the haplotype data as a tree, because the causal variant is embedded within the coalescent process describing the genealogy of the haplotypes under study [32].

Consider, for example, the incidence matrix for the haplotype data reported in Table 9. Columns correspond to 12 diallelic SNPs and rows identify the unique haplotypes and we assume there are 800 haplotypes in total. Alleles at each SNP position are coded as 0 for the major allele (i.e., the most frequent in the population) and 1 for the minor allele. Data are compatible with a rooted phylogeny if and only if, for any two SNPs (or columns) in the incidence matrix, the pattern of (01, 10, 11) is not present. An explanation of this constraint is that since the infinitely-many-sites model does not allow for back or recurrent mutation, the only way for these three gametic types to exist in the sample is for at least one recombination event to have occurred between the two sites [33]. Therefore, the use of the perfect phylogeny model requires both observations of little or no recombination in DNA segments [1–3,34], and the infinitely-many-sites assumption of population genetics.

**Table 9:**
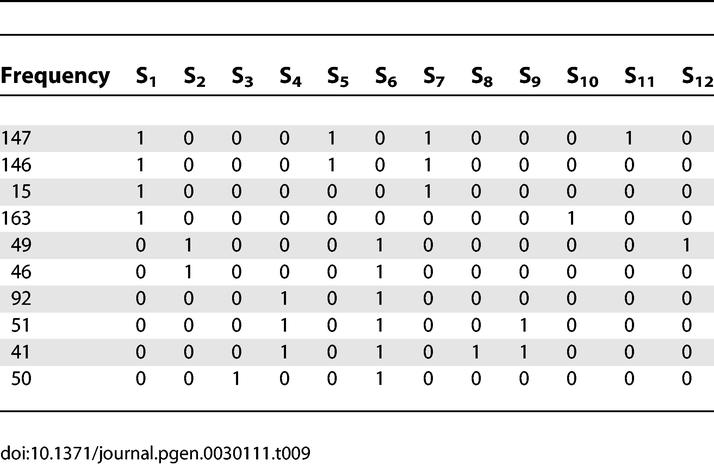
Incidence Matrix for Ten Distinct Haplotypes Together with Their Frequencies, Consisting of 12 SNPs (S1–S12), Where 0 Is the Major Allele and 1 Is the Minor Allele

**Figure 9:**
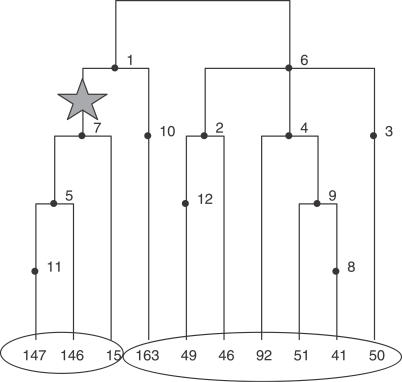
The Gene Tree Consistent with the Haplotypes in the Incidence Matrix of Table 6 Labels 1–12 refer to mutations S1–S12. At the bottom of each branch we report the multiplicity of each observed haplotype in the sample.

It is possible to construct a gene tree when the perfect phylogeny condition is true for all pairs of SNPs of a study sample using, for example, Gusfield's algorithm [35,24]. Figure 9 shows the gene tree for the haplotypes in Table 9. The nodes in the tree correspond to mutations that have generated the segregating sites and the gene tree is rooted at the haplotype with all major alleles. Mutations are ordered on the tree according to their relative age. If the causal mutation is embedded between SNPs 1 and 7, all descendant haplotypes of that lineage will inherit it and, therefore, we expect that most case haplotypes are among the 308 haplotypes that correspond to the first three branches of the tree (first three lines of Table 9). Thus, in the region of the disease locus, a sample of case haplotypes tend to have a more-recent shared ancestry than do control haplotypes, because many of them share a recent disease mutation. Note, however, that sporadic cases due to phenocopies, dominance, and epistasis introduce substantial noise in the phenotype–haplotype relationship, which influences the relative frequencies of nonpenetrant case haplotypes carried by unaffected controls and control haplotypes carried by affected cases.

The proposed method can be applied to a single candidate region, multiple candidate regions, and to fine-scale mapping. Recent studies suggest that recombination events occur preferentially outside genes [34,36]. Thus, in the case of single or multiple candidate regions we assume that each gene lies in a region of high LD. Within each region, then, we assume, as described above, a coalescent model of evolution and the infinitely-many-sites model and represent each gene with a separate tree. For fine-scale mapping, the chromosomal segment can be divided into a number of gene trees with boundaries determined by loci in which the perfect phylogeny assumption is violated. Details of how this is achieved are given in the following section.

### Splitting a chromosomal region into perfect phylogenies.

A rooted perfect phylogeny (PP) assumption poses the constraint that, for any two SNPs in the incidence matrix, not all three combinations (01, 10, 11) exist. Recombination and back or parallel mutation leads to the possible existence of all three combinations. We have developed an R routine based on the algorithm of Lenhard [37] that scans a chromosomal region consisting of *m* markers and splits the region into sub-regions that satisfy the PP condition. In particular, starting from SNP *S*_1_, it checks the PP condition between SNPs *S*_1_ and *S*_2_. If the condition is true, it checks the condition between pairs *S*_1_ and *S*_3_, and *S*_2_ and *S*_3_. If the condition is not valid for SNPs *S*_ 1_ and *S*_ 3_, then SNPs *S*_ 1_ and *S*_ 2_ form a gene tree and the procedure is repeated starting from SNP *S*_ 3_. The same happens if the condition is valid for *S*_ 1_ and *S*_ 3_, but not for *S*_ 2_ and *S*_ 3_. If the condition is true for both pairs, the algorithm checks the PP assumption pairwise between SNPs *S*_ 4_ and *S*_ 1_– *S*_ 3_. Generally, if the pattern of (01, 10, 11) is identified between SNPs *S*_i_ and *S*_j_ (for every *i* < *j*), but not identified for any pairs between *S_k_*_1_ and *S_k_*_2_ (for *i* ≤ *k*1 < *k*2 < *j*), then SNPs *S_i_* − *S_j_*_− 1_ form a perfect phylogeny, and the procedure is repeated starting from SNP *S_j_*. However, note that this algorithm leads to only one of the possible tree configurations for the chromosomal region under study, since using different SNPs as a starting point may result in different tree configurations.

### Bayesian partition model.

As mentioned earlier, the proposed method splits the haplotype space into disjoint clusters on the basis of haplotype similarity, with the number of clusters unknown, a priori. To measure the closeness of one haplotype to another, we adopt a distance that has an evolutionary interpretation, with sequences sharing a cluster depending on the time to their most recent common ancestor. Thus, the distance metric is based on the relative ages of the mutations in the sample or on the order with which the mutations have arisen in the haplotype sample, which is provided by the topology of the gene tree. Any SNP set selected as cluster centres can therefore be time ordered, and we assign haplotypes to clusters according to the relative ages of the centres. Suppose, for example, that SNPs 4, 5, and 7 of Figure 9 are selected as cluster centres. SNP 7 is older than SNP 5, and SNP 4 is on a different branch, implying that a haplotype carrying mutation 4 cannot carry mutation 5 or 7. Starting with SNP 5, we assign the haplotypes that correspond to the first two branches of the tree (namely, the first two haplotypes in Table 9) as members of this cluster. The only member of the cluster with SNP 7 as centre is the third haplotype, because, although the first two haplotypes carry mutation 7, they have been already allocated to a cluster. The seventh, eighth, and ninth haplotypes are allocated to a separate cluster with centre SNP 4, and all remaining haplotypes are assigned to a hypothetical “null” cluster, which can be interpreted as a baseline risk group. Therefore, the choice of the centres defines the way that haplotypes are assigned to their clusters. Given the centres, every haplotype is deterministically allocated to the cluster with the closest centre, using the metric above.

Haplotypes within each cluster have cluster-specific risks of disease, which are assumed to be exchangeable and to come from some simple distribution. As mentioned earlier, this is intended to capture the fact that haplotypes that are similar to each other in the region of a putative causal mutation are likely to be associated with similar risks of disease. An MCMC algorithm is developed to obtain posterior samples of quantities of interest, averaging over the space of possible partitions. In particular, we are interested in the posterior distribution of the number of clusters and the posterior probability that each SNP is chosen as a cluster centre. For example, in the extreme scenario of a fully penetrant variant that is among the set of typed markers, we expect a high posterior probability of having only two clusters, namely, the cluster with the causal variant as cluster centre and the “null” cluster.

### Model specification.

For simplicity, let us first consider the case in which the haplotype data form a single perfect phylogeny, as in Table 9. Assume that the haplotype space is currently partitioned into *n*_c_ = *n*_clust_ + 1 independent clusters (*n*_c_ includes the “null” cluster, while *n*_clust_ is the number of SNPs selected as cluster centres). A convenient approach to parameterising the space of possible partitions is to introduce an indicator vector **γ**, with **γ** = (γ_1_,…γ_*n*_SNP__), with γ*_k_* in {0,1}, *k* = 1,…,*n*_SNP_, such that γ*_k_* = 1 if the *k*th SNP is selected as cluster centre and γ*_k_* = 0 otherwise, where *n*_SNP_ is the number of SNPs in the dataset. That is, there is a one-to-one map from the space of possible partitions to the sample space of **γ**.

Next, *y_ij_* in {0,1} is the disease status indicator of haplotype *i* = 1,…,*n_j_* in cluster *j* = 1,…,*n*_c_ *.* The vector of responses for cluster *j* is denoted by ***D*** *_j_* = (*y*_1*j*_,*y*_2*j*_,…*y_n_j___j_*) and let ***D*** = {***D*** *_j_*, *j* = 1,…,*n*_c_}. Each *y_ij_* is assumed to have a Bernoulli distribution with parameter θ*_j_*, the disease risk associated with cluster *j*. The Bayesian formulation is completed by specifying priors on the parameters **θ** and **γ**. We assume a uniform prior on **γ**, i.e., the probability of each cluster configuration is equal to 1/2^*n*_SNP_^. Note that this induces a probability distribution on the number of cluster centres; the probability of having *n*_clust_ cluster centres is equal to 
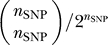
 . Cluster-specific risks are then given a conjugate Beta distribution with parameters α and β. This choice of prior distributions leads to computational advantages. In particular, the posterior distribution of **γ** is proportional to the product of its prior distribution and the marginal probability of the data where the latter is available analytically as

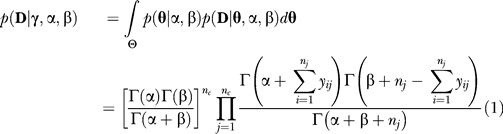
where Γ denotes the Gamma function and Θ = 
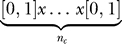
 .

Similarly, the full conditional distribution of the risk parameters is readily sampled from, as it is available in closed form

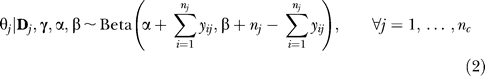



In the case of *n*_tr_ perfect phylogenies or trees (i.e., when we split the regions into separate windows or where we consider more than one candidate region), an extra layer is added in the hierarchy of the model, since the partition **γ** is now conditional on the tree *T* selected to cluster haplotypes. In particular, we specify a uniform prior on the trees, so that, a priori, each tree is equally likely to contain the putative mutation (recall that the underlying rationale is to exploit between marker LD around a putative causal variant, independent of the extent of LD or number of markers corresponding to each tree). The joint prior distribution of a gene tree *T* and a partition **γ** is given by

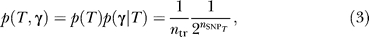
where 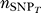 denotes the number of segregating sites in gene tree *T*. Details of the proposed MCMC algorithm are given later on.

Note that instead of assuming a uniform prior on the trees, we could use a more informative prior distribution. For example, if the average marker density is large, we would expect recombination to break the perfect phylogeny condition frequently, resulting in several trees with a small number of SNPs and a few trees with a larger number of SNPs. In this case, it might be more appropriate to use a prior distribution that favours trees with a small number of markers, such as the geometric distribution.

Upon convergence, from the posterior sample of partitions we obtain the posterior probability that the causal mutation is embedded in the ancestry of each of the gene trees. The mean and standard deviation of the posterior risk associated with each unique haplotype in the sample are also obtained. Furthermore, we estimate the Bayes factor in favour of association at each marker, which is given by the ratio of the posterior odds to prior odds [26]. The prior of each SNP being a cluster centre is evaluated by simulation using Equation 3. Finally, we use the location of the SNP with the highest marginal posterior probability of being a cluster centre as an estimate of the location of the susceptibility allele.

### Construction of 95% credible intervals.

**Figure 10:**
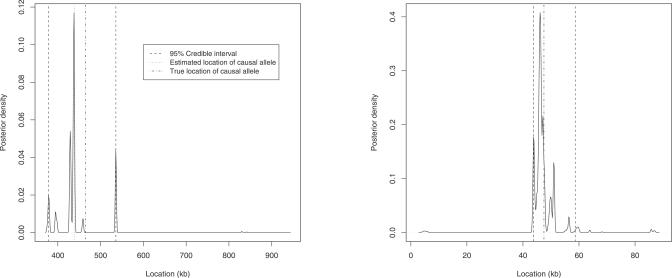
Posterior Density of Location of Causal Allele and 95% Credible Intervals Credible intervals (95%) of causal location for two datasets simulated with 1.8 and 2.4 GRR(Aa) and all other simulation parameters at default values. The credible intervals are 150 kb and 15 kb wide, respectively.

For the proposed method, it is straightforward to construct credible intervals for the estimated location of the causative SNP. At each iteration of the MCMC algorithm, we obtain an estimate of the causal location by averaging the locations of the markers currently selected as cluster centres. Thus, upon convergence, we obtain a posterior distribution of locations, from which a credible interval can be constructed. Under the default simulation scenario, in 43 out of the 50 replicates, the 95% credible interval contained the true causal locus. Figure 10 shows the posterior densities of the putative location of the causative variants, together with 95% credible intervals for two datasets simulated with 1.8 and 2.4 GRR(Aa), with all other simulation parameters set at their default values. The credible intervals are 150 and 15 kb wide, respectively.

### Sensitivity to prior specification.

To assess the sensitivity of the results to prior specification, we assigned Gamma(10,10) hyperpriors to the parameters α and β of the Beta prior on disease risks. We then ran the model for 100 different datasets simulated with variable recombination rate, additive disease model with GRR(Aa) 2, SNP density 1 kb, 200 cases and controls, MAF 5%, and MAF of the causal SNP 5%–7%, and obtained an average distance of 274.66 kb (22.98 kb standard error), compared to an average distance of 237.49 kb (25.17 kb standard error) of the standard model. As expected, in this case, the average distance was higher than before, since we allowed for more sources of uncertainty. However, both models resulted in a similar average number of clusters.

### The MCMC algorithm.

Considering the case of a single perfect phylogeny, we use a M-H step to sample from the full conditional distribution of the vector **γ** given the data. Namely, we consider two possible moves in the partition space: (1) Birth step: adding a cluster centre. (2) Death step: deleting a cluster centre.

Each move entails selecting a SNP at random, and proposing to change 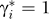 , if the current γ*_i_* = 0 (birth) or 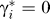 otherwise. Thus, the proposal distribution *q*(**γ** ^*^|**γ**) is simply 1/*n*_SNP_. Given the cluster centres, the observed haplotypes are deterministically allocated to the haplotype clusters according to the time in which they share a common ancestor in the genealogy with the cluster centres (as described earlier). Since we assume a conjugate Beta distribution for **θ**, the acceptance probability simplifies to min(1,Bayes factor(γ^*^,γ)) = min(1,*p*(*D*|**γ** ^*^,α,β)/*p*(*D*|**γ**,α,β)), where the marginal probability is calculated using Equation 1.

In the case of *n*_tr_ perfect phylogenies, we need an extra MCMC step in which we sample the tree containing the putative mutation. At each MCMC iteration, we now have two M-H steps: (1) Change partition step: sample a new partition from the posterior distribution of the number of clusters and the cluster centres without changing the current gene tree. (2) Update tree: sample a new tree and a new partition from their joint posterior distribution.

The first M-H step is the same as the one used in the case of a single gene. For the second M-H step, assuming a uniform prior on the trees, the joint prior distribution of a gene tree *T* and a partition **γ** is given by Equation 3. In particular, we first sample a tree from the *n*_tr_ possible trees with probability 1/*n*_tr_, and then each SNP in the tree has a 0.5 probability of being a cluster centre. Therefore, the proposal move in the tree and the partition space is equal to Equation 3. This leads to an acceptance probability for the second M-H sampler that again only involves the Bayes factor in favour of the proposed partition over the current partition. In summary, the MCMC algorithm is:

1. Randomly choose a gene tree and initialize the partition (and therefore the *T* and **γ** parameters) within the tree. Calculate the marginal probability of the model under partition **γ**.
2. Within the current tree *T,* choose randomly one SNP, i.e., SNP *S_i_,* and switch its γ*_i_* indicator from zero to one (and vice versa), thus proposing a new partition **γ** ^*^. Allocate haplotypes to the cluster with the “closest” centre. Calculate the marginal probability of the data under the proposed partition **γ** ^*^ using equation 1.
3. Evaluate the Bayes factor in favour of **γ** ^*^, and calculate the acceptance probability. If accepted, set **γ** = **γ** ^*^.
4. Randomly choose a gene tree *T* ^*^, and propose a move for **γ** given *T* ^*^ by generating **γ** ^*^ from the prior. Allocate haplotypes to the cluster with the “closest” centre. Calculate the marginal probability of the data under the proposed partition **γ** ^*^ using Equation 1.
5. Evaluate the Bayes factor in favour of **γ** ^*^, and calculate the acceptance probability. If accepted, set *T* = *T* ^*^ and **γ** = **γ** ^*^.
6. Repeat steps 2–5 until convergence.

## Supporting Information

Figure S1 Results from BETA, Margarita, HAPCLUSTER, and Fisher's Exact Test from a Case-Control Study under the Default Scenario Marginal posterior probability of association from BETA (top left), Bayes factor in favour of association at each marker from BETA (top right), *p*-values from Margarita and Fisher's exact test (bottom left), and posterior density of location from HAPCLUSTER (bottom right), where the dot on the x-axis indicates the position of the susceptibility mutation. (This legend applies to Figures S1–S14.)
(141 KB EPS)

Figure S2 Results from BETA, Margarita, HAPCLUSTER, and Fisher's Exact Test from a Case-Control Study under the Default Scenario (140 KB EPS)

Figure S3 Results from BETA, Margarita, HAPCLUSTER, and Fisher's Exact Test from a Case-Control Study under the Default Scenario (141 KB EPS)

Figure S4 Results from BETA, Margarita, HAPCLUSTER, and Fisher's Exact Test from a Case-Control Study under the Default Scenario (141 KB EPS)

Figure S5 Results from BETA, Margarita, HAPCLUSTER, and Fisher's Exact Test from a Case-Control Study under the Default Scenario (141 KB EPS)

Figure S6 Results from BETA, Margarita, HAPCLUSTER, and Fisher's Exact Test from a Case-Control Study under the Default Scenario (141 KB EPS)

Figure S7 Results from BETA, Margarita, HAPCLUSTER, and Fisher's Exact Test from a Case-Control Study under the Default Scenario (141 KB EPS)

Figure S8 Results from BETA, Margarita, HAPCLUSTER, and Fisher's Exact Test from a Case-Control Study under the Default Scenario (140 KB EPS)

Figure S9 Results from BETA, Margarita, HAPCLUSTER, and Fisher's Exact Test from a Case-Control Study under the Default Scenario (142 KB EPS)

Figure S10 Results from BETA, Margarita, HAPCLUSTER, and Fisher's Exact Test from a Case-Control Study under the Default Scenario (141 KB EPS)

Figure S11 Results from BETA, Margarita, HAPCLUSTER, and Fisher's Exact Test from a Case-Control Study under the Default Scenario (141 KB EPS)

Figure S12 Results from BETA, Margarita, HAPCLUSTER, and Fisher's Exact Test from a Case-Control Study under the Default Scenario (141 KB EPS)

Figure S13 Results from BETA, Margarita, HAPCLUSTER, and Fisher's Exact Test from a Case-Control Study under the Default Scenario (141 KB EPS)

Figure S14 Results from BETA, Margarita, HAPCLUSTER, and Fisher's Exact Test from a Case-Control Study under the Default Scenario (141 KB EPS)

Table S1 Performance Comparison Performance comparison of BETA with HAPCLUSTER and single locus analysis for MAF of the causal SNP equal to 5%–7% and number of case/control genotypes equal to 200, and for different SNP densities, minor allele frequencies, and GRRs. Results obtained over 100 repeats under each scenario.
(34 KB DOC)

Table S2 Performance Comparison Performance comparison of BETA with HAPCLUSTER and single locus analysis for MAF of the causal SNP equal to 10%–15% and number of case/control genotypes equal to 200, and for different SNP densities, minor allele frequencies, and GRRs. Results obtained over 100 repeats under each scenario.
(34 KB DOC)

## Abbreviations

ARG: ancestral recombination graph
GRR: genetic relative risk
LD: linkage disequilibrium
MAF: minor allele frequency
MCMC: Markov Chain Monte Carlo
M-H: Metropolis-Hastings
PP: perfect phylogeny
SNP: single nucleotide polymorphism
