# Supplementary material for: Genetic Association Mapping via Evolution-Based Clustering of Haplotypes
Source: PLoS Genet. 2007 Jul 6;3(7):e111. doi: 10.1371/journal.pgen.0030111 (PMC1913101; doi:10.1371/journal.pgen.0030111)
Supplement: Supplementary file 1 [file pgen.0030111.st001.doc]

Table S1: Performance comparison of BETA with HAPCLUSTER and single locus analysis for causal allele frequency equal to 5-7% and number of case/control genotypes equal to 200, and for different SNP densities, minimum allele frequencies, and GRRs. Results obtained over 100 repeats under each scenario.

| Marker spacing (kb) | 1 | 1 | 1 | 1 | 3 |
| --- | --- | --- | --- | --- | --- |
| Minimum allele frequency (%) | 5 | 5 | 1 | 1 | 5 |
| Genetic relative risk (Aa) | 2 | 3 | 2 | 3 | 2 |
| Causal allele frequency (%) | 5-7 | 5-7 | 5-7 | 5-7 | 5-7 |
| BETA:  average distance (kb)  (standard error) | 237.49  (25.17) | 70.82  (9.22) | 196.37  (21.62) | 67.07  (8.85) | 190.88  (22.25) |
| BETA (fixed number of clusters):  average distance (kb)  (standard error) | 192.10  (22.43) | 65.25  (8.74) | 192.47  (21.04) | 58.18  (8.26) | 198.47  (22.99) |
| Minimum p-value:  average distance (kb)  (standard error) | 205.38  (22.11) | 86.07  (14) | 202.73  (21.73) | 54.64  (7.76) | 199.04  (22.74) |
| HAPCLUSTER:  average distance (kb)  (standard error) | 243.47  (23.63) | 62.58  (11.13) | 204.53  (21.61) | 66.60  (12.08) | 231.10  (24.47) |
