# Supplementary material for: Genetic Association Mapping via Evolution-Based Clustering of Haplotypes
Source: PLoS Genet. 2007 Jul 6;3(7):e111. doi: 10.1371/journal.pgen.0030111 (PMC1913101; doi:10.1371/journal.pgen.0030111)
Supplement: Supplementary file 2 [file pgen.0030111.st002.doc]

Table S2: Performance comparison of BETA with HAPCLUSTER and single locus analysis for causal allele frequency equal to 10-15% and number of case/control genotypes equal to 200, and for different SNP densities, minimum allele frequencies, and GRRs. Results obtained over 100 repeats under each scenario.

| Marker spacing (kb) | 1 | 1 | 1 | 1 | 3 |
| --- | --- | --- | --- | --- | --- |
| Minimum allele frequency (%) | 5 | 5 | 1 | 1 | 5 |
| Genetic relative risk (Aa) | 2 | 3 | 2 | 3 | 2 |
| Causal allele frequency (%) | 10-15 | 10-15 | 10-15 | 10-15 | 10-15 |
| BETA:  average distance (kb)  (standard error) | 82.21 (14.71) | 19.35 (1.98) | 120.31 (20.21) | 18.52 (2.37) | 88.36 (16.20) |
| BETA (fixed number of clusters):  average distance (kb)  (standard error) | 79.09 (13.86) | 19.10 (1.87) | 93.09 (17.47) | 22.35 (3.56) | 93.21 (16.34) |
| Minimum p-value:  average distance (kb)  (standard error) | 107.27 (19.60) | 20.34 (1.87) | 107.46 (19.50) | 21.88 (2.43) | 88.95 (15.91) |
| HAPCLUSTER:  average distance (kb)  (standard error) | 108.88 (19.26) | 22.64 (6.22) | 101.21 (18.83) | 19.94 (3.81) | 89.67 (16.46) |
